# Acellular porcine placental membranes as a novel biomaterial for tissue repair applications

**DOI:** 10.3389/fbioe.2025.1606615

**Published:** 2025-06-18

**Authors:** Gustavo Henrique Doná Rodrigues Almeida, Luan Stefani Lima, Mariana Sversut Gibbin, Beatriz Lopomo, Rafael Oliveira Bergamo, Raquel Souza da Silva, Giovanna Vitória Consani Santos, Bruna Gomes Silva, Isabela Paulillo D’Onofrio, Henrique dos Santos, Lediane Pedroso Silva, Tais da Silva, Henrique Lança Fuzeti, Bianca Fuzeti Candian, Thais Naomi Gonçalves Nesiyama, João Victor Damin, Claudio Guilherme de Assis Oliveira, Lucas Paulo Jacinto Saavedra, Guilherme Henrique Gonçalves de Almeida, Douglas Lopes de Almeida, Jaqueline de Carvalho Rinaldi, Francielle Sato, Mauro Luciano Baesso, Luzmarina Hernandes, Flávio Vieira Meirelles, Rose Eli Grassi Rici, Durvanei Augusto Maria, Paulo Cezar de Freitas Mathias, Ana Claudia Oliveira Carreira

**Affiliations:** ^1^ Postgraduation Program of Anatomy of Domestic and Wild Animals, School of Veterinary Medicine and Animal Science, University of São Paulo, SãoPaulo, Brazil; ^2^ Department of Physics, State University of Maringá, Maringá, Brazil; ^3^ Department of Morphological Sciences, State University of Maringá, Maringá, Brazil; ^4^ Department of Veterinary Medicine, Faculty of Animal Science and Food Engineering, University of São Paulo, Pirassununga, Brazil; ^5^ Department of Biotechnology, Genetics and Cell Biology, State University of Maringá, Maringá, Brazil; ^6^ Center for Natural and Human Sciences, Federal University of ABC, Santo André, Brazil

**Keywords:** placenta, porcine, biocompatibility, biomaterial, decellularization

## Abstract

Biological dressings derived from the extracellular matrix (ECM) of human placental tissues have proven effective in treating complex skin wounds and other anatomical sites, offering potential for new therapeutic applications. However, the use of human tissues is limited by ethical and biosafety concerns, restricting large-scale production. To address this, biomaterials from placentas of livestock animals offer a cost-effective, accessible alternative without harming animal welfare. Given pigs’ large-scale production, short gestation periods, and abundant material availability, this study aimed to produce, characterize, and validate acellular biomembranes derived from decellularized porcine allantochorion for tissue repair. Placental fragments from Duroc sows were decellularized using a protocol involving immersion and orbital shaking in 0.1% SDS and 0.5% Triton X-100, followed by low-frequency ultrasonication. Accelularity was confirmed by total genomic DNA quantification and H&E and DAPI staining for nuclear visualization. Membrane structure and composition were analyzed using histological, immunohistochemical methods, and scanning electron microscopy. Spectroscopic analyses detected physicochemical changes in placental ECM, and biomechanical testing assessed membrane strength and stiffness. Biological functionality was validated through *in vitro* cell viability and adhesion assays with canine endothelial progenitor cells and L929 murine fibroblasts. *In vivo* biocompatibility was tested by subcutaneously implanting the biomaterial in rats for histopathological evaluation. Results showed efficient decellularization, with preserved ECM structure. The scaffolds were cytocompatible, supporting cell adhesion and high viability. *In vivo* testing revealed no immune rejection, confirming biocompatibility and biodegradability. In conclusion, acellular porcine placental biomembranes have the necessary characteristics to be explored as scaffolds for tissue engineering and novel repair therapies.

## 1 Introduction

Therapeutic approaches based on bioderivatives from extraembryonic tissues, particularly human placentas, for the repair and regeneration of injured tissues have demonstrated significant outcomes. The versatility of these tissues enables their wide-ranging application across various clinical fields (Deus et al., 2020; Moore et al., 2017). Research has explored the regenerative potential of these tissues in diverse niches, from wound healing in skin and ophthalmic applications to the regeneration of more rigid tissues such as bone and cartilage (Protzman et al., 2023). Both crude extracts from placental tissues and isolated, purified bioproducts, such as mesenchymal stem cells, placental extracellular matrix (ECM) components, and exosomes in conditioned media, have been shown to effectively modulate the tissue microenvironment and support the repair of damaged areas. (Arki et al., 2023; Biswas et al., 2023; Silini et al., 2015). This biotechnological and translational potential has transformed the placenta from a temporary, disposable organ at the end of pregnancy into a rich source of materials for regenerative medicine (Choi et al., 2013; Silini et al., 2015). Unlike other tissues, placental tissues do not require invasive procedures for harvesting, nor the use of cadaveric tissues, making them a sustainable and bioavailable resource for the production of novel therapeutic bioproducts (Gujjar et al., 2024; Roy et al., 2022).

Placental tissues possess a range of biological properties that significantly enhance their regenerative bioactivity. These include notable pro- and anti-angiogenic properties, promoting a balance in new blood vessel formation; anti-inflammatory, immunomodulatory, anti-fibrotic, and antimicrobial activities; as well as increased levels of cell proliferation and differentiation (Dubus et al., 2022; Hortensius et al., 2016; Ramuta et al., 2021; Wassmer and Berishvili, 2020). Interestingly, bioscaffolds derived from the extracellular matrix of these tissues retain these beneficial properties, along with biomechanical characteristics that support effective interactions between the scaffolds and cellular components (Rameshbabu et al., 2020). Furthermore, unlike matrices from other tissues, placental matrices are highly biocompatible due to the placenta being a naturally immunoprivileged organ. This enables better integration with the host’s adjacent tissues, resulting in minimal immune rejection and enhanced bioactivity (Hussein et al., 2016; Shi et al., 2015).

Among the several applications of placental tissues and matrices for tissue repair, most studies focus on dermatological therapies, addressing large wounds or those complicated by associated comorbidities such as infections and diabetes (Alizadeh et al., 2022; 2024; Gholipourmalekabadi et al., 2018). Research has shown that acellular derivatives of human amniotic membrane, chorion, and umbilical cord possess regenerative bioactivity, with commercial products like BIOVANCE^®^ and EpiFix^®^ already available for use (Letendre et al., 2009; Mahmoudi-Rad et al., 2013; Z. Wu et al., 2018). However, as noted, the production of these human-derived biodressings involves several bioethical and biosafety challenges that complicate and increase the cost of the process (Almeida G. H. D. et al., 2022; De Kanter et al., 2023). In light of this, scaffolds derived from placentas of other animals, particularly livestock animals such as cattle, sheep, and pigs, have attracted attention as promising alternatives for new regenerative therapies in both medical and veterinary settings (Carvalho et al., 2021; Leonel et al., 2017; Lobo et al., 2016). Some studies have demonstrated that non-human placental scaffolds can support the culture of several cell types from different species, confirming their translational potential (Baracho Trindade Hill et al., 2020; Barreto et al., 2022; Barreto et al., 2019; Oliveira et al., 2023). Furthermore, *in vivo* biocompatibility studies using murine models have verified the non-immunogenicity of acellular murine and bovine placental matrices, indicating that these materials can be effectively used as biografts for tissue repair and regeneration (Carvalho et al., 2021).

Considering this, the production of biodressings from porcine placentas presents significant biological and commercial potential. Since pigs are widely raised for human consumption, the collection of placentas, naturally discarded after birth, does not cause any additional harm or waste (Adhikari et al., 2018; Khan et al., 2023; Tarafdar et al., 2021). The epitheliochorial placenta contains a highly vascularized region known as the allantochorion or chorioallantoic membrane, which is in intimate contact with the uterus and plays a key role in maternal-fetal communication (Johnson et al., 2021; Kaczynski et al., 2023). This structure exists within a highly pro-angiogenic, proliferative, and immunomodulatory microenvironment, suggesting that its extracellular matrix is imbedded with growth factors, cytokines, and microvesicles with potent biological activity (Capella-Monsonís et al., 2023). Therefore, the aim of this study was to produce, characterize, and validate the biological potential of decellularized porcine allantochorion scaffolds, highlighting their biotechnological potential in tissue engineering.

## 2 Materials and methods

### 2.1 Placental tissue acquisition and preparation

**FIGURE 1:**
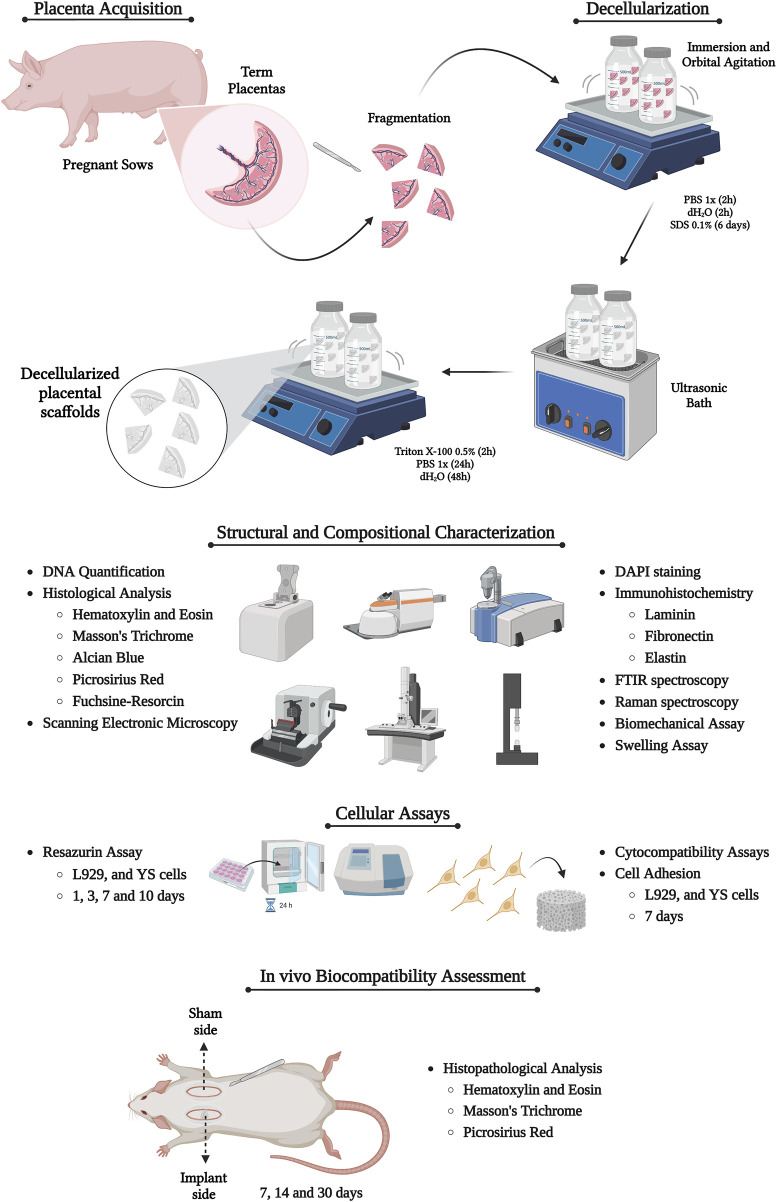
Experimental design of the study, highlighting the production and characterization of placental acellular scaffolds. Created with BioRender.com.

Term placentas (n = 10) from pregnant Duroc sows were collected from University of São Paulo, Pirassununga campus, Pirassununga, São Paulo State, Brazil with the institution’s deed of donation and authorization for use. The samples were initially separated, then pooled to ensure randomness and capture the inherent variability across specimens for the subsequent assays. The samples were washed, kept in ice and transported to the Laboratory of Tissue Engineering of the Department of Surgery of the School of Veterinary Medicine and Animal Science from the University of São Paulo. This research was performed according to institutional ethics committee regulations of the University of São Paulo (CEUAx protocol no. 9963170523). The experimental design is summarized in Figure 1.

### 2.2 Acellular membranes production and characterization

#### 2.2.1 Decellularization

At first, porcine placentas were washed with distilled water and PBS 1x to remove any dirty or residual blood. Then, the amniotic membrane and the umbilical cord were dissected and removed, leaving only the chorioallantois membrane (CAM). The membranes were segmented in 2 cm^2^ size pieces, which were submitted to decellularization. The following protocol was used to produce the acellular scaffolds: 1) first washing–the samples were washed with deionized water (dH2O) and PBS 1X to remove the residual blood for 2 h each; 2) decellularization part I–sodium dodecyl phosphate (SDS) 0.1% for 6 days; 3) ultrasonication–the samples were immersed in dH2O and underwent for1 cycle of 1 min in the ultrasonic bath (Sonicator Ultrasonic Misonix Processor XL); 4) decellularization part II–Triton X-100 0.5% for 2 h; 5) final washing - 24 h of dH_2_O and 2 h of PBS. Placental samples were stored in PBS 1x at 4°C for further analysis. Five samples of each group (Native and Decellularized) were used for every assay described below.

#### 2.2.2 Scaffolds sterilization

After decellularization, the scaffolds were thoroughly washed with dH_2_O for 48 h to remove any detergent residues. In a laminar flow hood, the matrices were then fragmented and immersed in a series of alcohol solutions with progressive and regressive concentrations (70%, 80%, 90%, 100%, 90%, 80%, and 70%), with each concentration maintained for 5 min. Following this, the scaffolds were rinsed with 1X PBS containing 2% antibiotics (Penicillin-Streptomycin 10,000 μg/mL, LGC Biotecnologia, Cotia, Brazil). To ensure further sterilization, the matrices were exposed to UV light for 5 min and stored for later analysis. To assess the sterility of the scaffolds, they were immersed in antibiotic-free DMEM medium (Sigma, St. Louis, MO, United States) and incubated for 72 h in a cell culture incubator.

#### 2.2.3 DNA content quantification

For total genomic DNA quantification, the Bio Gene^®^ K204-4 (BioGene, Recife, Brazil) was used according to the manufacturer’s specifications. Fragments from native and decellularized samples were digested 56°C by the action of Proteinase K. The fragments were filtered and analyzed by spectrophotometry at 260 nm (Nanodrop, Thermo Scientific, Waltham, MA, United States).

#### 2.2.4 4,6-Diamidino-2-Fenilindole (DAPI) staining

Samples were embedded in Tissue Plus O.C.T. (Fisher Healthcare, Houston, TX, United States), frozen and microsectioned using a cryostat (CM1860 model, Leica Biosystems, Baden-Wurttemberg, Germany). The slides were stained with DAPI solution (1:10,000) at room temperature without light for 10 min. Then, they were washed with PBS 1x for analysis using fluorescent microscopy (Nikon ECLIPSE 80I, CADI FMVZ-USP, Tokyo, Japan).

#### 2.2.5 Histological analysis

Native and placental samples were fixed in 4% paraformaldehyde (PFA) for 48 h, dehydrated using crescent alcohol (70, 80, 90, and 100%), diaphanized in xylol, and embedded in paraffin. 5 µm microsections were stained with Hematoxylin & Eosin (HE) to observe general morphological structure and nuclei presence; Masson’s trichrome for total collagen content evaluation; Alcian blue (pH = 2.5) to evaluate total GAGs general content; Weigert’s fuchsin-resorcin to observe elastic fibers content and Picrosirius Red to evaluate collagen distribution. Slides were photographed and analyzed using a light microscope (Nikon ECLIPSE 80I, CADI FMVZ-USP). For morphometric quantification, it was used the occupied area were quantified in five semiseries slices per animal, totaling 10 fields at the magnification of 20 times with the software ImageJ version 8.0.

#### 2.2.6 Immunohistochemistry analysis

Slices were rehydrated in a citrate buffer in the microwave for 1 min for antigen retrieval. The citrate buffer was prepared by dissolving citric acid monohydrate to a final concentration of 10 mM in distilled water. The pH was then adjusted to 6.0 using 1 M sodium hydroxide, and the volume was brought up to 1 L. Then, the endogenous peroxidase blockage was performed with 3% hydrogen peroxide in distilled water for 40 min in the dark. For the non-specific protein interaction blockage, 2% bovine serum albumin (BSA) in PBS was used. The primary antibodies chosen were anti-fibronectin (#Ab2413, 1:100, Abcam, Cambridge, UK), anti-laminin subunit α2 (#PA1-16730, 1:200; Invitrogen) and anti-elastin (#Ab9519, 1:100, Abcam) and the secondary antibodies were IgG anti-mouse/anti-rabbit (#K800; Dako, CA, United States). The antibodies incubated overnight 4°C. The reaction was detected by Dako Advance HRP (#K6068; Dako) followed by DAB (#k3468; Dako) revelation, according to the manufacturer’s instructions. Slides were photographed and analyzed using a light microscope (Nikon ECLIPSE 80I, CADI FMVZ-USP). For morphometric quantification, it was used the occupied area were quantified in five semiseries slices per animal, totaling 10 fields at the magnification of 20 times with the software ImageJ version 8.0.

#### 2.2.7 Scanning electronic microscopy (SEM)

Placental samples were fixed in Karnovsky solution (2.5% glutaraldehyde and 4% paraformaldehyde in a buffered 0.1 M sodium cacodylate) for 48 h and dehydrated in alcohol concentrations for 5 min each. The samples were dried in in a supercritical point device (LEICA EM CPD 300^®^) and coated with gold (EMITECH K550^®^, Quorum Technologies, United Kingdom). Then, the samples were photographed under a scanning electron microscope (LEO 435 VP^®^, Oberkochen, Germany).

#### 2.2.8 Fourier transform infrared spectroscopy (FTIR) analysis

Fourier transform infrared spectroscopy (FTIR) was used to assess ECM physic-chemical composition of native and decellularized placental samples. The technique was performed in a Bruker Vertex 70v FTIR spectrometer (Bruker Optik GmbH, Ettilingen, Germany) with an attenuated total reflectance (ATR) accessory located at the Department of Physics from the State University of Maringá, Paraná, Brazil. The spectrum of each sample (n = 5), between 4000 and 400 cm^−1^, is an average of three measurements with 128 scans and 4 cm^−1^ of spectral resolution. The spectra were vector normalized using OPUS software 8.7 SP2 and the experiments were conducted at room temperature.

#### 2.2.9 Raman spectroscopy analysis

Raman spectroscopy was performed on the same groups, but on samples dried at the critical point to enhance spectral data collection and maintain sample integrity. Spectra were recorded using a Senterra Confocal Raman Microscope (Bruker Optik GmbH, Ettlingen, Germany), equipped with a 785 nm laser excitation source at 100 mW power, focused onto the sample with a ×20 magnification lens at room temperature. The spectral range covered was from 1750 to 400 cm^-1^, with a spectral resolution between 9 and 15 cm^−1^. Each spectrum represents the average of three acquisitions, with each acquisition being an average of 30 individual spectra, and an integration time of 15 s per spectrum. Data were normalized using a normalization vector through the OPUS software.

#### 2.2.10 Biomechanical evaluation

The biomechanical properties characterization of the developed placental biomembranes was assessed through a tensile test, which was carried out at the Bioengineering Laboratory, University of São Paulo, Ribeirão Preto campus, Brazil. Five native and five decellularized placental segments measuring 7 × 4 cm were placed on computerized universal testing machine (EMIC model DL 1000) and subjected to a tensile load of 500 N until failure. The pre-load speed was set at 20 mm/min, while the testing speed was 30 mm/min. Maximum tensile force, maximum elongation, and stiffness were evaluated.

#### 2.2.11 *In vitro* swelling assay

The *in vitro* swelling capacity assay protocol was adapted from (Asgari et al., 2021). Initially, the placental scaffolds were lyophilized and weighed to determine their dry mass (W_0_) prior to the experiment. After weighing, the samples were immersed in 1X PBS and incubated at 37°C. At intervals of 30 min, 1, 2, 3, 4, 24, 36, and 48 h, the scaffolds were removed, the excess water was removed using filter paper, and the swollen samples were weighed to determine their final mass after each time point (n = 5). The swelling capacity ratio was calculated using the following equation:

$$\frac{Swelling\;ratio\;\left(\%\right)=W\left(f\right)-W\left(o\right)\;}{W\left(o\right)}\;x\;100$$

### 2.3 Cytocompatibility analysis

#### 2.3.1 Cell viability assay

To evaluate the cytotoxic potential of placental biomembranes, the resazurin colorimetric assay was conducted as described in (Almeida et al., 2024a). Three cell types were used in the assays: canine Yolk sac-derived (YS) cells, which were isolated, characterized, and generously provided by Professor Ana Claudia Oliveira Carreira from the University of São Paulo (Fratini et al., 2016) and murine L929 fibroblasts, kindly donated by Professor Maria Isabel de Souza Aranha Melaragno’s laboratory from the Federal University of São Paulo (UNIFESP). These cells were cultured in DMEM high glucose medium, supplemented with 10% fetal bovine serum (FBS, Gibco, United Kingdom) and antibiotics, and incubated at 37°C under 5% CO_2_. After an 18-h incubation period, the cells were trypsinized and seeded at a concentration of 2 × 10^3^ cells/mL onto the placental membranes in 24-well plates. Then, 1 mL of resazurin solution (0.14 mg/mL in PBS; Thermo Fisher, Waltham, MA, United States) was added to each well and incubated with the 0.5 cm^2^ scaffolds for 1, 3, 7, and 10 days. At each time point, 200 μL aliquots were collected and stored in a 96-well plate in the refrigerator for subsequent analysis. After the experimental period, the samples were measured using a spectrophotometer (µQuant–Bio-Tek Instruments, INC) at an optical density of 540 nm. Cells cultured under 2D conventional conditions in a well plate were used as the experimental control.

#### 2.3.2 Cell adhesion assay

To assess the ability of the placental biomembranes to induce cell adhesion, 2 × 10^4^ YS, L929, and HaCat cells were seeded onto the scaffolds for 7 days under the same conditions described previously. After this period, the seeded scaffolds were fixed and analyzed using Scanning Electron Microscopy (SEM) to assess the cell-matrix interaction.

### 2.4 *In Vivo* biocompatibility assessment

#### 2.4.1 Animals and ethical issues

The animal experiments were carried out at the State University of Maringá (UEM), Paraná, Brazil, following approval from the Ethics Committee on the Use of Animals (protocol n° 55061190624/2024). Male Wistar rats (n = 15) from the UEM Central Vivarium were used in the study. The animals were housed individually in cages within the Animal Facility of the Department of Morphological Sciences (DCM-UEM), under controlled conditions: temperature of 22°C ± 1°C, relative humidity of 50% ± 10%, and a 12-h light/dark cycle. They were provided with food and water *ad libitum*. All procedures complied with the European Community Council Directive on the use of animals for scientific purposes.

#### 2.4.2 Scaffolds subcutaneous implantation

For the evaluation of the biocompatibility of the biomembranes, a subcutaneous implant model was carried out according to (Almeida et al., 2024b). The animals were first anesthetized using a combination of ketamine and xylazine (0.03 mg/mL). The dorsal area of the animals was shaved and cleaned in preparation for the surgical procedure. A 1 cm^2^ sample of placental scaffolds was then implanted subcutaneously on the right side of the animals' dorsal region, with the left side serving as a Sham control. A small incision, no larger than 1 cm, was made to access the tissue and perform the subcutaneous implant. Post-surgery analgesia was administered using Tramadol (20 mg/mL diluted in Ringer’s lactate) between 12 and 24 h after the procedure to ensure the animals experienced minimal pain or discomfort. The animals were housed in numbered cages under controlled acclimatization conditions. At 7-, 14-, and 30-day post-surgery, the animals were euthanized (five animals per time point) using an overdose of ketamine (300 mg/kg) and xylazine (30 mg/kg).

#### 2.4.3 Histopathological analysis

After euthanasia, the implants along with the surrounding skin tissue were collected and processed for histological analysis, as previously described. For histopathological evaluation, tissue sections were stained with Hematoxylin and Eosin (HE) to assess general tissue morphology and inflammatory response, Masson’s Trichrome to evaluate collagen deposition and organization, and Picrosirius Red for qualitative analysis of collagen fiber types under polarized light. Biocompatibility was assessed using a semiquantitative scoring system adapted from ISO 10993-6 guidelines. The following parameters were evaluated: intensity of inflammatory cell infiltration (including lymphocytes, macrophages, and multinucleated giant cells), fibrous capsule thickness, and degree of collagen deposition. Each parameter was graded on a scale from 0 to 4, where 0 = absent, 1 = minimal, 2 = mild, 3 = moderate, and 4 = severe. A cumulative biocompatibility score was calculated by summing the individual values, enabling comparison between the two experimental groups. Picrosirius Red staining was used qualitatively to observe the organization and distribution of collagen fibers. Under polarized light, type I collagen appeared as thick red-orange birefringent fibers, while type III collagen appeared as thin green-yellow fibers, providing complementary information on matrix organization. Ten random fields per sample were analyzed at ×200 magnification. Quantitative data were expressed as mean ± standard deviation and statistically analyzed using an unpaired Student’s t-test, with significance set at p < 0.05.

### 2.5 Statistical analysis

The normality of the data was first assessed using the Shapiro-Wilk test. The Mann-Whitney test was applied for quantitative data, with results presented as mean ± standard deviation (SD). Principal component analysis (PCA) was performed for spectroscopic assays, followed by one-way ANOVA to compare the means. Statistical significance was set at p < 0.05, and Tukey’s *post hoc* test was applied where appropriate. All statistical analyses were conducted using GraphPad Prism 8.0 (GraphPad Software, Inc., San Diego, CA, United States).

## 3 Results

### 3.1 Decellularization efficiency evaluation

**FIGURE 2:**
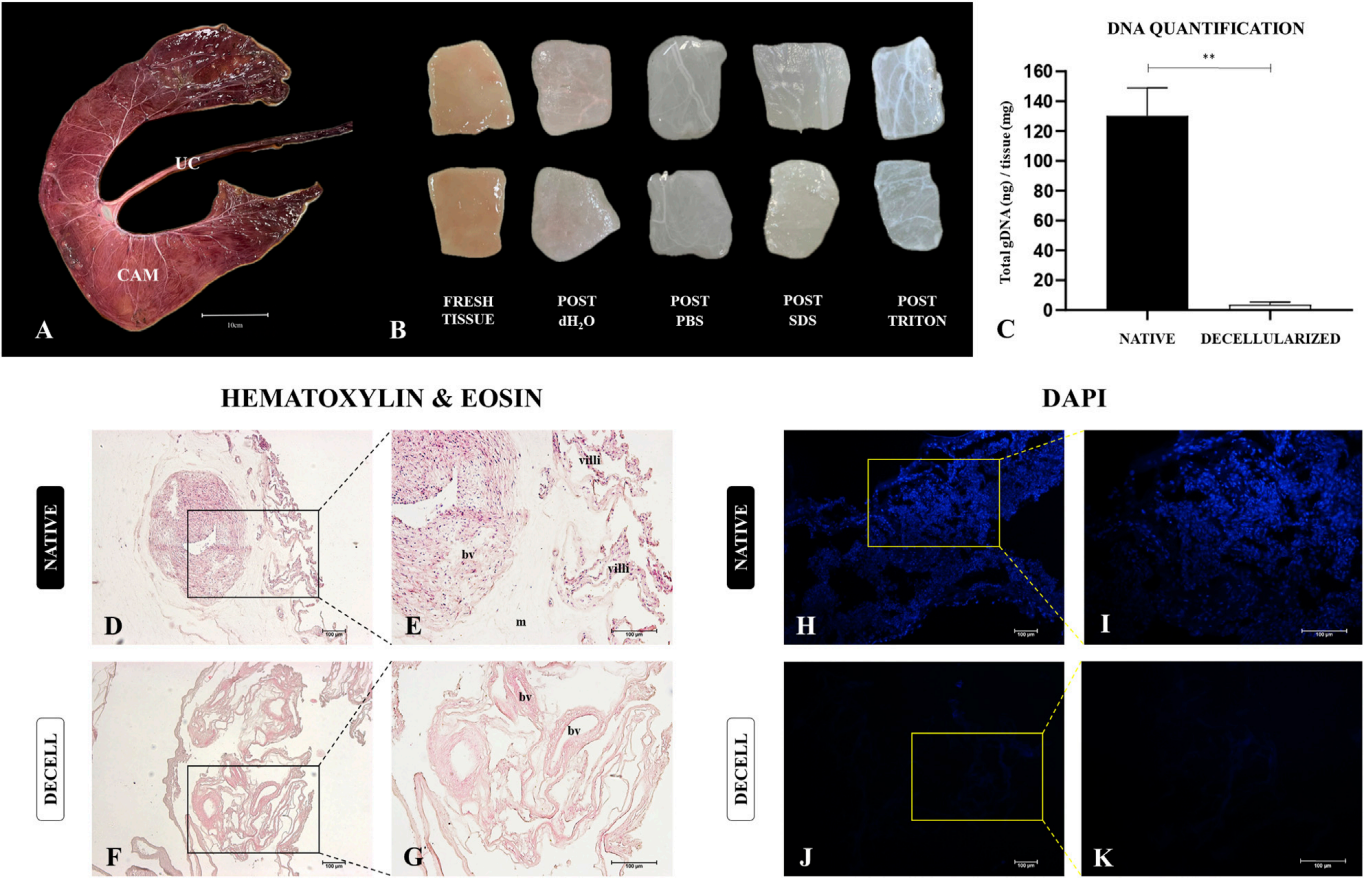
Production and decellularization efficiency evaluation of porcine decellularized placental biomembranes (n = 5 per group). Photodocumentation of a dissected porcine epitheliochorial placenta, highlighting the chorioallantoic membrane (CAM) **(A)**. CAM macroscopic aspects after each step of the decellularization process, showing the samples gradual whitening **(B)**. Total genomic DNA quantification of placental tissues before and after the decellularization **(C)**. Hematoxylin and Eosin and DAPI staining showing the nuclei absence compared to the native tissue **(D–K)**. **p < 0.05 compared to the native group.

Structurally, the porcine placenta is classified as diffuse epitheliochorial, with the allantochorion, or Chorioallantoic membrane (CAM), representing the most abundant, thick, and vascularized portion of the fetal membranes. It is also in direct contact with the maternal endometrium (Klisch and Schraner, 2021). Given these characteristics, the CAM was selected for the production of porcine placental scaffolds due to its higher extracellular matrix content, as well as its highly vascularized microenvironment, which lies at the interface of maternal-fetal communication (Figure 2A). Macroscopically, the decellularization process was confirmed by the gradual discoloration of the tissue as the decellularizing solutions progressed, ultimately resulting in complete whitening of the tissue (Figure 2B). Quantification of total genomic DNA demonstrated a 97.3% reduction in DNA content compared to native samples, indicating the effectiveness of the proposed protocol in removing nucleic acids (Figure 2C). Furthermore, both H&E and DAPI staining revealed the absence of nuclei in the decellularized samples, corroborating the DNA quantification results (Figures 2D–K). H&E staining also showed the preservation of key structural features of the porcine CAM, suggesting that the structural framework was largely intact following decellularization, with no clear signs of extracellular matrix degradation compared to control samples (Figures 2D–G).

### 3.2 Morphological and Structural characterization

**FIGURE 3:**
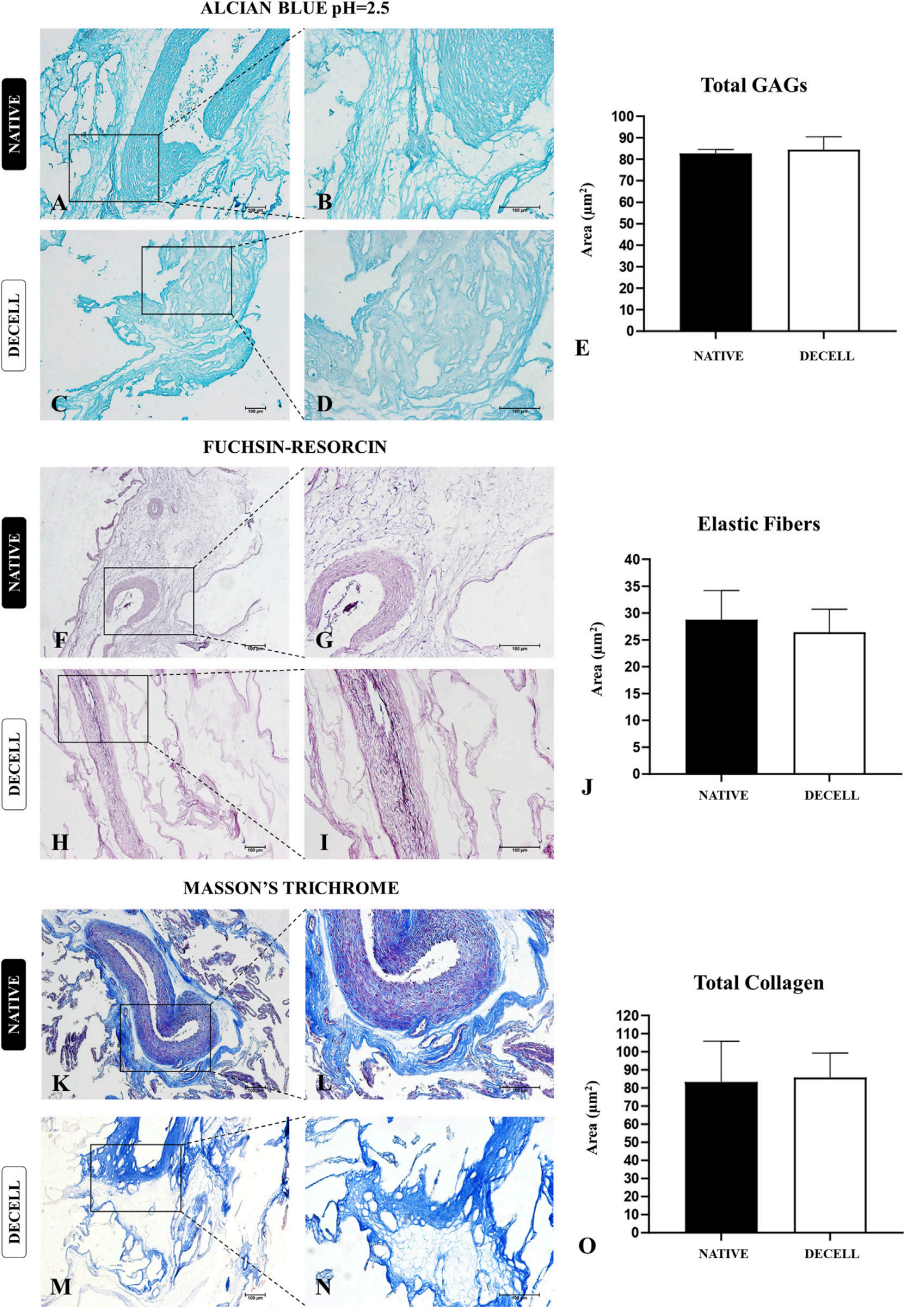
Structural evaluation of extracellular matrix components of native and decellularized porcine placental membranes (n = 5 per group). Alcian Blue staining for total glycosaminoglycans (GAGs) content **(A–D)** and total GAGs semiquantitative analysis **(E)**. Fuchsin-resorcin staining for elastic fibers content **(F–I)** and elastic fibers semiquantitative analysis **(J)**. Masson’s trichrome staining for total collagen content **(K–N)** and total collagen content semiquantitative analysis **(O)**.

The preservation of the key components of the placental extracellular matrix (ECM) ensures that the produced scaffold retains the three-dimensional structure of the native tissue, as well as the molecular composition of the tissue microenvironment, facilitating proper interaction with cellular populations (Almeida et al., 2022b). To assess this, a morphological and ultrastructural characterization was performed on both the native placental tissue and the acellular membranes, to evaluate the integrity of the primary extracellular matrix proteins. Initially, histological analyses (Figure 3) were conducted to evaluate and quantify, through specific staining, total glycosaminoglycans (Figures 3A–E), elastic fibers (Figures 3F–J), and total collagen (Figures 3K–O). In terms of total glycosaminoglycans (GAGs), the semiquantitative analysis of the area stained with Alcian Blue (Figure 3E) showed no significant differences between the native samples (82.628 ± 1.938) and decellularized samples (84.490 ± 5.926). Regarding the elastic fibers, which are responsible for the elasticity and flexibility of the placental tissue and are highlighted in dark purple by Weigert’s Fuchsin-Resorcin staining (Figures 3F–J), no significant differences were observed between the native (28.77 ± 5.400) and the decellularized membranes (26.44 ± 4.270).

**FIGURE 4:**
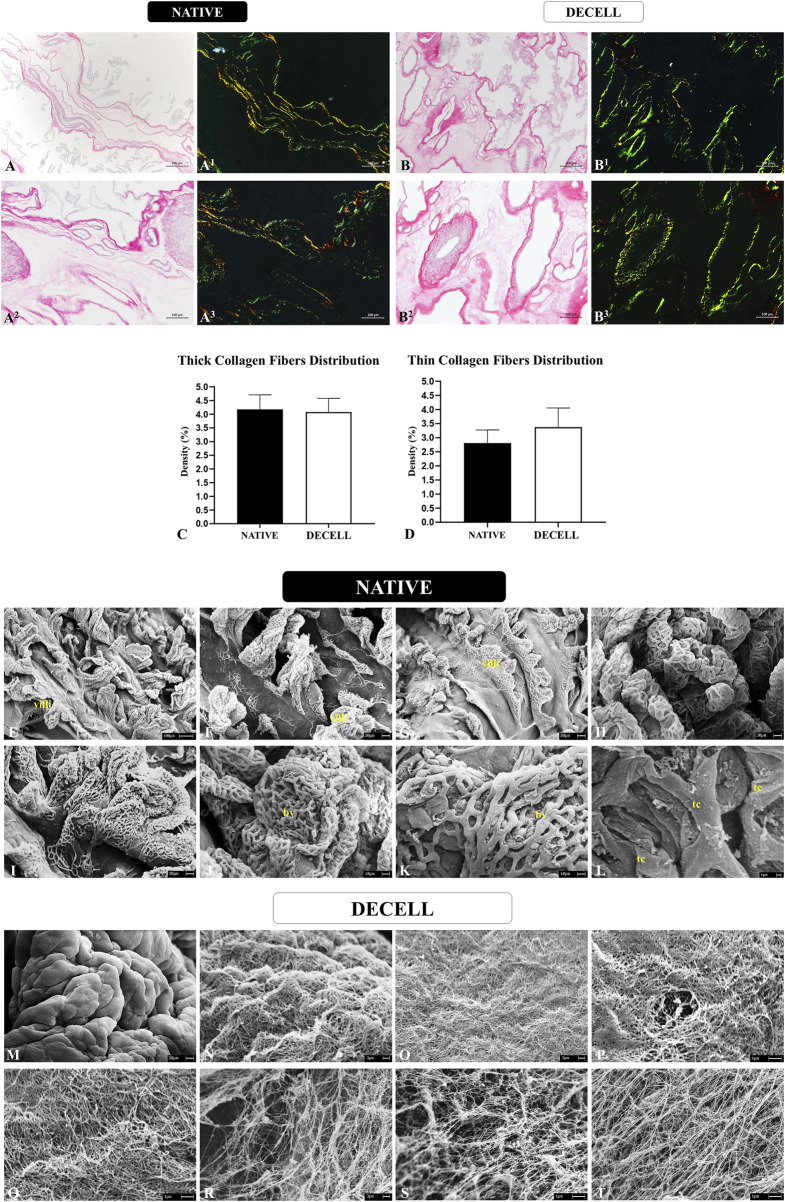
Collagen fibers distribution pattern evaluation of native and decellularized porcine placental membranes (n = 5 per group). Non-polarized and polarized Picrosirius Red staining to differentiate thick collagen fibers stained in reddish and yellowish tones from thin collagen fibers stained in greenish tones in native **(A-A** ^**3**^ **)** and decellularized **(B-B** ^**3**^ **)** samples. Semiquantitative analysis of thick and thin collagen fibers distribution **(C,D)**. Ultrastructural analysis by scanning electronic microscopy of native **(E–L)** and decellularized porcine placental membranes **(M–T)**. villi (chorionic villi), bv (blood vessel), tc (trophoblastic cell).

Considering that collagen is the most abundant component present in the porcine chorioallantoic membrane, a more in-depth analysis was conducted to assess the preservation of both the composition and the three-dimensional structure of these components. The morphometric analysis, carried out by quantifying the area occupied by total collagen stained blue in Masson’s trichrome staining (Figures 3K–O), revealed no significant changes between the native samples (83.28 ± 22.540) and decellularized samples (85.73 ± 13.50). To evaluate the integrity of both mature and immature collagen fibers, Picrosirius Red staining was performed, which differentiates thick, more mature collagen fibers with reddish and yellowish tones, while thinner, more immature collagen fibers stain in greenish hues (Figures 4A,B). The quantitative analysis also showed no significant changes in either fiber type, suggesting that the heterogeneous framework of collagen fibers withstood the decellularization process (Figures 4C,D). For the thicker collagen fibers, the morphometric distribution values for the native samples were 4.177 ± 0.5314, while for the decellularized samples, they were 4.080 ± 0.497. For the thinner collagen fibers, the morphometric distribution values for the native samples were 2.810 ± 0.4613, while for the decellularized samples, they were 3.373 ± 0.682.

The three-dimensional structure of the placental scaffolds was further evaluated using scanning electron microscopy to assess the integrity of the placental membranes following the decellularization process (Figures 4E–T). In the native tissue, a high density of chorionic villi is observed, accompanied by an intricate vascular network responsible for maternal-fetal exchanges. These structures are predominantly supported by the basal membrane, which is in direct contact with the placental mesenchyme, the area with the highest concentration of extracellular matrix in the allantochorion (Figures 4E–L). These chorionic and vascular structures are no longer present in the decellularized samples, as the detergents effectively removed the cellular components, leaving only the fiber framework. A smooth surface is visible at lower magnification (Figure 4M), indicating the absence of villi. At higher magnification, a complex and intertwined network of fine and thick fibers forms a well-organized mesh (Figures 4N–T). No evidence of fiber degradation or disorganization is seen, suggesting the preservation of the three-dimensional placental connective tissue microarchitecture. These ultrastructural observations, associated with the histological findings, strongly support the conclusion that the placental scaffolds retained their structural integrity after decellularization.

**FIGURE 5:**
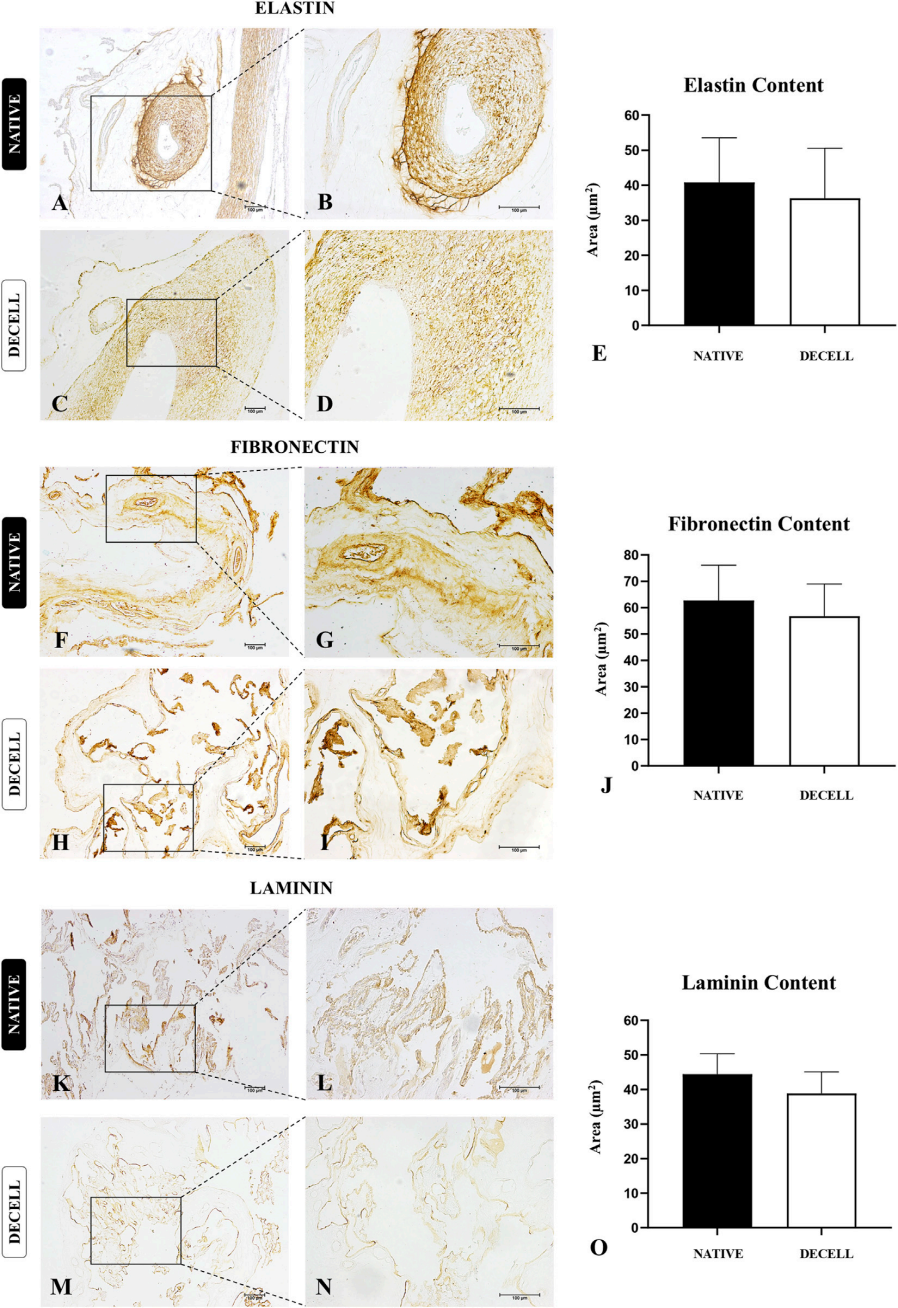
Immunohistochemical analysis of key extracellular matrix proteins of native and decellularized porcine placental membrane samples (n = 5 per group). Immunostaining of elastin **(A–D)**, fibronectin **(F–I)** and laminin **(K–N)**. Semiquantitative comparative analysis of elastin **(E)**, fibronectin **(J)** and laminin **(O)** content between native and decellularized placental samples.

To complement the morphological data, immunohistochemical analyses of three key extracellular matrix proteins, elastin, fibronectin, and laminin, were performed (Figure 5). The morphometric analysis of the area occupied by elastin (Figure 5E), the primary protein constituent of elastic fibers, showed no significant differences between the native samples (40.82 ± 12.68) and decellularized samples (36.30 ± 14.26). In the allantochorion, elastin was predominantly localized in blood vessels, with a smaller presence in the placental stroma (Figures 5A–D). Regarding fibronectin, a glycoprotein essential for cell adhesion and communication between the intra- and extracellular microenvironments, the morphometric analysis of the area occupied (Figure 5J) also revealed no significant differences between the native samples (62.67 ± 13.38) and decellularized samples (56.79 ± 12.15). The distribution of fibronectin in the stroma was similar in both groups, suggesting that this critical protein remained largely intact after the decellularization process (Figures 5F–I). The morphometric analysis of the area occupied by laminin (Figure 5O), another adhesive glycoprotein found in higher amounts in the basal membrane, also showed no significant differences between the two groups. However, a slight decrease was observed in the decellularized samples (38.83 ± 6.257) compared to the native samples (44.42 ± 5.925). Considering that laminin consists of multiple subunits, and this analysis focused on the most abundant subunit, α2 (Sainio and Järveläinen, 2020), the decellularization process may have affected each subunit differently, potentially accounting for the slight decrease. Nevertheless, the distribution pattern of laminin in the basal membrane of the chorionic villi and blood vessels remained intact, suggesting that both the structural and compositional integrity of the scaffolds were preserved (Figures 5K–N).

### 3.3 ECM physic-chemical composition characterization

**FIGURE 6:**
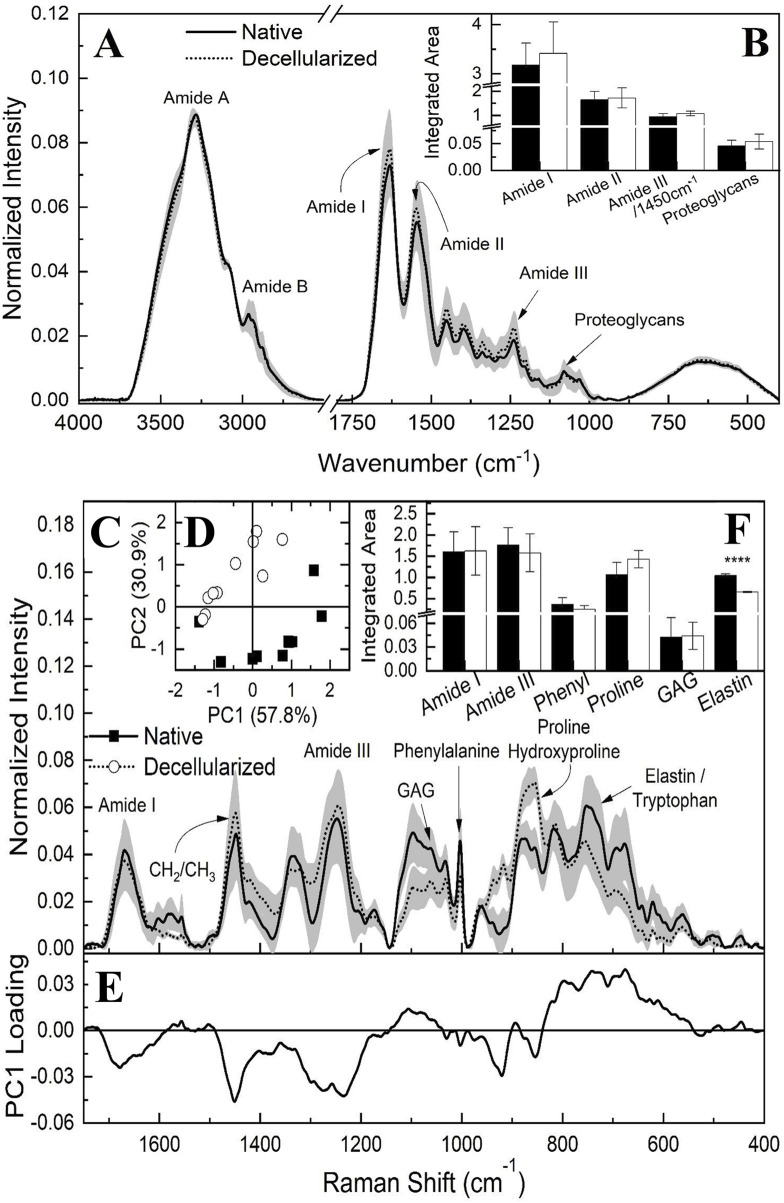
FTIR-ATR average spectrum of native and decellularized porcine placental membranes (n = 5 per group) with the respective standard deviation (SD) highlighted in gray **(A)**. Integrated band areas associated to triple helical structure of collagens (ratio between amide III and 1,450 cm^-1^), elastin content, proteoglycan content and collagen (Amide I and II) of native and decellularized placental samples **(B)**. Raman spectroscopy average spectrum of native and decellularized porcine placental membrane samples (n = 5 per group) with the respective standard deviation (SD) highlighted in gray **(C)**. Score plot of principal component analysis (PCA) for native and decellularized samples **(D)**. PC1 loading plot highlighting spectral differences between groups **(E)**. Integrated band areas associated to the content of Amide I, Amide III, phenylalanine, proline/hydroxyproline, glycosaminoglycans, and elastin **(F)**. ****p < 0.0001 compared to the native tissue.

In previous studies of our group, vibrational spectroscopy using FTIR-ATR and Raman techniques was employed to characterize and assess the physicochemical alterations in native and decellularized samples of porcine ovaries, fallopian tubes, and uterine horns (Almeida et al., 2023; Almeida et al., 2024a; Almeida et al., 2024b). The bands corresponding to amides are particularly prominent in the FTIR-ATR spectra (Figure 6), as they are directly associated with the collagen content of the material. Specifically, Amide I can serve as a marker for the secondary structure of proteins, while Amide II may be linked to the hydration of protein structures and could serve as an indicator of collagen self-assembly (Sohutskay et al., 2020; Wang et al., 2014). Regarding Amide III, it may correlate with the triple helical structures of collagen, particularly when comparing the vibrational ratio of this mode with the band attributed to CH_2_ bond deformation centered at 1,450 cm^−1^ (Wang et al., 2014). Amides A and B are also potentially related to the protein structures that constitute the collagen molecule, with bands centered around 3,280 cm^−1^ and 2,962 cm^−1^, respectively. Additionally, FTIR analysis reveals bands around 1,080 cm^−1^, which indicate the presence of covalently bound sulfated glycosaminoglycans (GAGs) attached to the core proteins (Nara et al., 2015; Wang et al., 2014).

To estimate the approximate content of each component, the areas under each vibrational mode were quantified through integration, after defining the regions corresponding to functional groups and drawing a baseline between the extreme points of each band (Figure 6B). The content of Amide I, Amide II, proteoglycans, and triple helical collagen structures (as indicated by the ratio between Amide III and 1,450 cm^−1^) did not show statistically significant differences, suggesting the absence of alterations in the extracellular matrix components detected by FTIR.

In addition to the FTIR data, Raman spectroscopy (Figure 6C) was also utilized to investigate the placental ECM components in both native and decellularized membranes. Among the bands identified, those associated with Amide I, centered at 1,665 cm^−1^, and Amide III, located around 1,245 cm^−1^, are particularly notable (Arezoo et al., 2021; Bergholt et al., 2019; Nara et al., 2015; Nguyen et al., 2013; Timchenko et al., 2017; Zhang et al., 2011). The band at 1,450 cm^−1^ corresponds to hydrocarbon vibrations typically associated with elastin and/or collagen (Arezoo et al., 2021), while the band at 1,062 cm^−1^ is linked to glycosaminoglycans (GAGs) (Arezoo et al., 2021; Bergholt et al., 2019; Nguyen et al., 2013). In the region around 1,005 cm^−1^, the spectrum corresponds to the aromatic rings of phenylalanine, while the band at 875 cm^−1^ is attributed to proline and hydroxyproline (Emami et al., 2021; Nguyen et al., 2013; Timchenko et al., 2017; Villaret et al., 2019). At 752 cm^−1^, the tryptophan spectrum is observed; however, this band may overlap with the elastin spectrum, which is typically identified in the range of 722–725 cm^−1^ (Ingle et al., 2025; Pielesz et al., 2019).

Principal component analysis (PCA) was conducted on the Raman spectra to identify spectral differences among the samples. Figure 6D displays the score plot, with PC1 accounting for the largest proportion of variance in the data (57.8%), followed by PC2 (30.9%). This analysis suggests a trend of differentiation between the samples, indicating potential spectral differences between the native and decellularized samples. However, the absence of a complete separation between the spectra implies that similar functional groups may still contribute to the data, possibly unaffected by the decellularization process. Figure 6E presents the loading plot for component PC1. The Amide III band significantly contributes to the spectral differentiation, followed by the CH_2_ and CH_3_ chains, as well as the regions associated with proline, hydroxyproline, and elastin. However, when the regions of the bands are marked and their respective areas integrated, as shown in Figure 6F, including Amide I, Amide III, phenylalanine, proline, hydroxyproline, GAGs, and elastin, with potential contributions from tryptophan, only the latter band displayed statistically significant differences. Thus, the Raman spectroscopy results align with the FTIR data. Finally, the reduction observed in the elastin-associated band was 36.9% when comparing the decellularized samples to the native samples.

### 3.4 Biomechanical properties assessment

**FIGURE 7:**
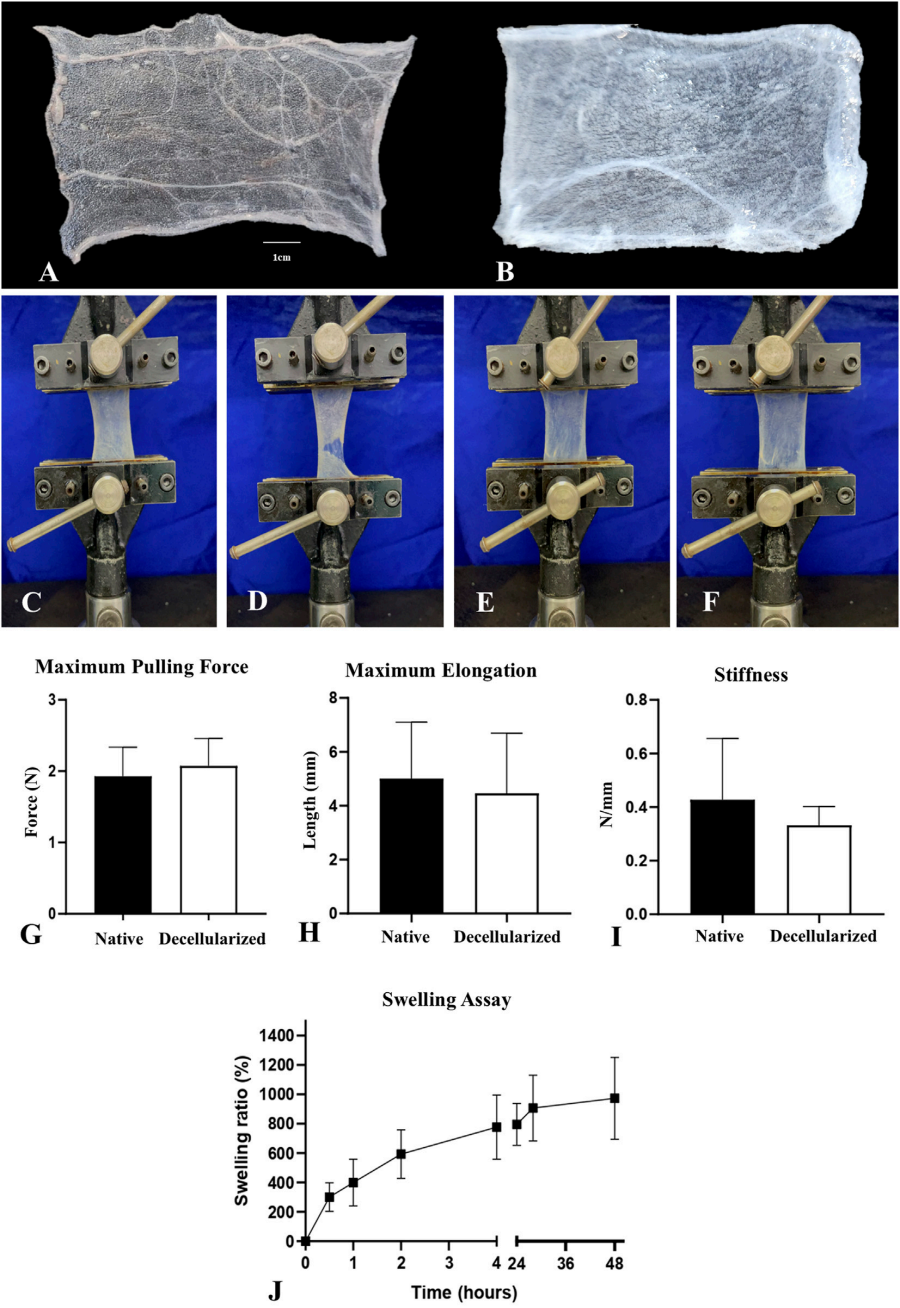
Biomechanical performance evaluation of native and decellularized porcine placental membranes (n = 5 per group). Representative images of native **(A)** decellularized **(B)** test specimens used in the assay. Photodocumentation of the biomechanical assay, demonstrating the positioning of the native and decellularized test specimens in the machine, respectively **(C,E)** and the rupture moment used to measure the mechanical parameters **(D,F)**. Maximum Pulling Force **(G)**, Maximum Elongation **(H)** and Stiffness **(I)** of native and decellularized placental samples. Swelling assay performed with decellularized placental membranes, demonstrating the swelling ratio in each evaluated period **(J)**.

The biomechanical performance analysis of the acellular placental biomembranes produced (Figure 7) is intrinsically linked to the structure of the extracellular matrix and directly impacts its functionality (Urbanczyk et al., 2020). By positioning the test specimens in the universal mechanical testing equipment until rupture (Figures 7C–F), three variables related to the mechanical properties of the tissues were evaluated: maximum pulling force (Figure 7G), maximum elongation (Figure 7H), and stiffness (Figure 7I). No statistical difference was observed between native and decellularized samples in the three parameters analyzed; however, with regard to stiffness, there was a slight decrease in the decellularized samples, although this change was not statistically significant. For maximum pulling force, the native samples had an average of 1.928 ± 0.4088 N, while the decellularized samples had an average of 2.075 ± 0.3811 N. For the maximum elongation parameter, the native samples showed an average of 5.006 ± 2.090 mm, and the decellularized samples had an average of 4.460 ± 2.232 mm. Regarding stiffness, the native membranes had an average of 0.4275 ± 0.2279, while the decellularized membranes showed an average of 0.3320 ± 0.07014. Additionally, a swelling test was conducted to assess the fluid retention capacity of the acellular membranes (Figure 7J). The results demonstrated that the volume of the samples significantly increased over 48 h, with the mass of the samples rising by more than 972% compared to the initial mass at 48 h. This indicates a high fluid retention capacity, which may be associated with the high porosity of the material.

### 3.5 *In vitro* cytocompatibility

**FIGURE 8:**
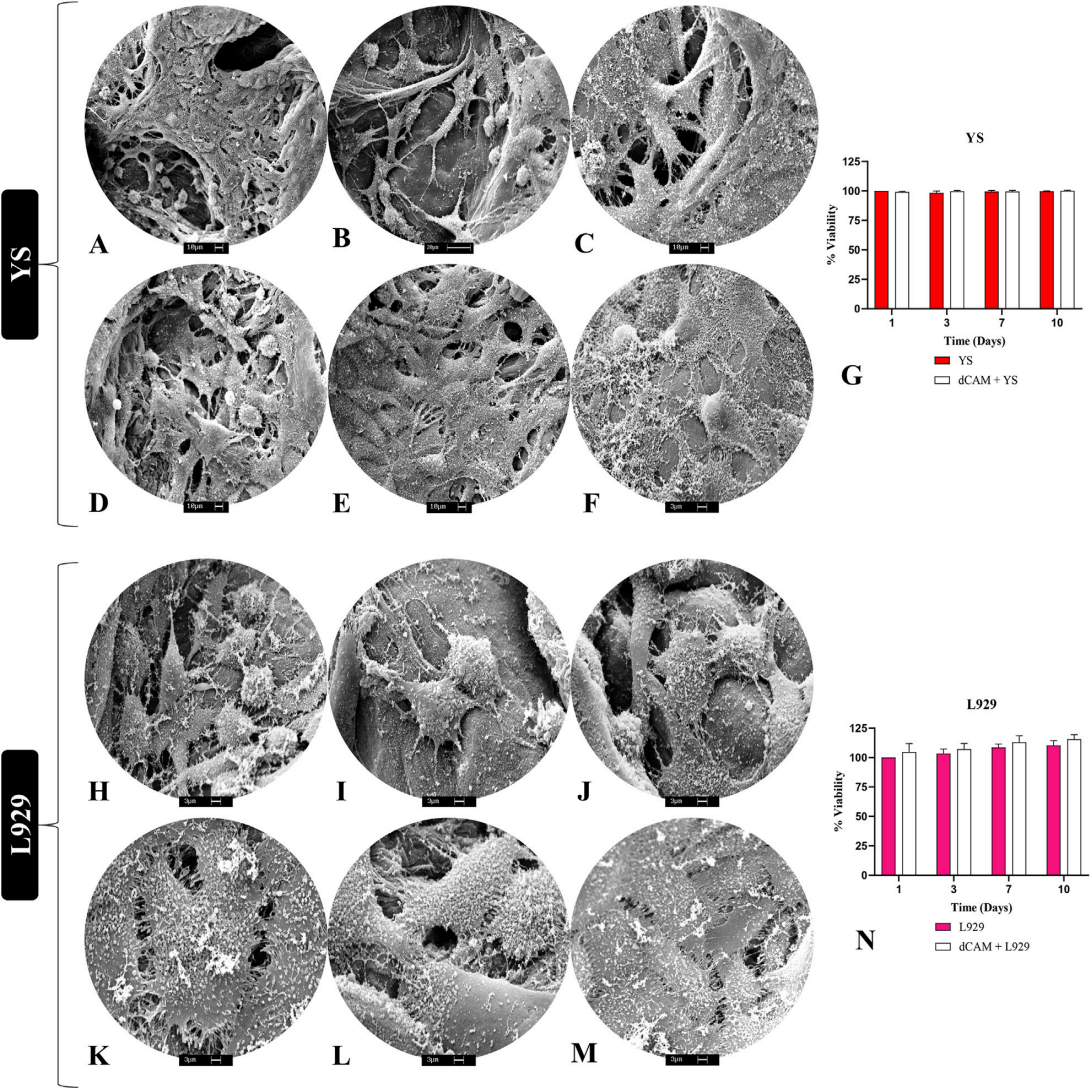
*In vitro* cytocompatibility evaluation of porcine placental decellularized membranes (n = 5 per cell type). Scanning electronic microscopy photodocumentation of YS cells **(A–F)** and L929 fibroblast cells **(H–M)** on the placental scaffolds ECM fibers, demonstrating their anchoring and adhesion. Viability assay of YS and L929 cells cultured on placental scaffolds (dCAM) **(G,N)**. Absorbance data were converted and expressed in viability percentage. YS vs. dCAM + YS and L929 vs. dCAM + L929.

To evaluate the biomembrane’s cytocompatibility, 2 cell types were employed to assess both their adhesion and interaction with the scaffolds, as well as cellular viability over a specified period (Figure 8). SEM analysis revealed that canine YS cells could adhere to porcine placental matrices (Figures 8A–F), exhibiting numerous extensions and a high cellular density, indicating a suitable cell-matrix interaction. Regarding viability, no statistically significant differences were observed between cells cultured in 2D on plastic and those cultured in 3D on the scaffolds, indicating the absence of cytotoxicity (Figure 8G). For L929 fibroblasts, cell interaction with the extracellular matrix was also evident through the presence of extensions, along with a notable abundance of rounded structures resembling extracellular vesicles, suggesting active cellular secretion (Figures 8H–M). Furthermore, the results demonstrated high cellular viability in fibroblasts cultured on the scaffolds, with slightly higher rates than those cultured in conventional 2D systems (Figure 8N). These findings suggest that the biomembranes can support the culture of various cell types across different species, indicating their potential for interspecies translational applications.

### 3.6 *In vivo* biocompatibility assessment

**FIGURE 9:**
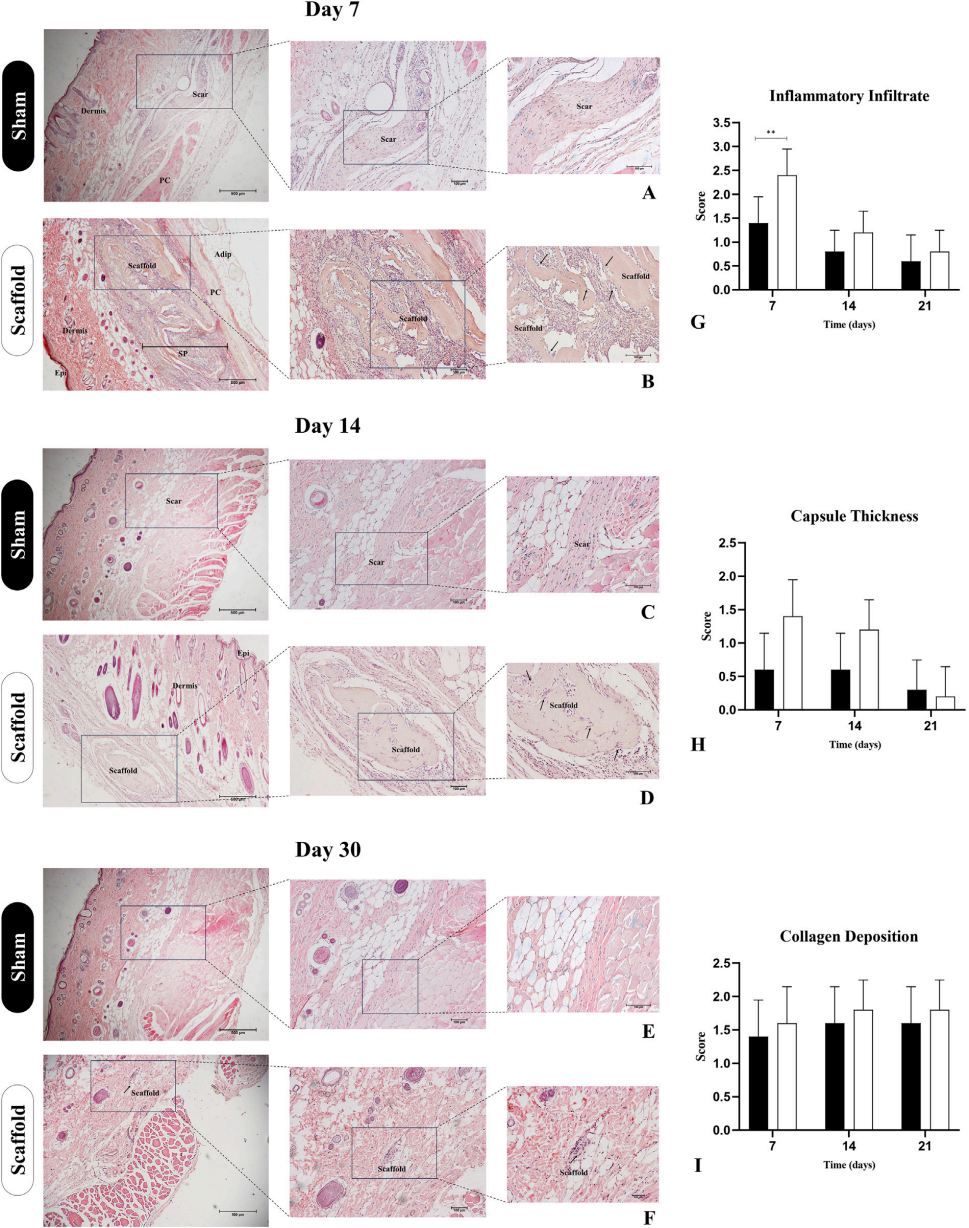
Histopathological assessment of decellularized placental membranes implanted in the subcutaneous tissue of immunocompetent rats for biocompatibility evaluation (n = 5 per group). Hematoxylin and Eosin staining of the implanted region on day 7 **(B)**, 14 **(D)** and 30 **(F)** and their respective Sham control groups **(A,C,E)**. Bar graphs represent semiquantitative scores (mean ± SD) for inflammatory infiltrate **(G)**, capsule thickness **(H)**, and collagen deposition **(I)**, comparing Scaffold and Sham groups at each timepoint. Epi (epidermis), SP (subcutaneous pocked), PC (*panniculus carnosus*), Adip (adipose tissue). Black arrows indicate the scaffolds borders with cell infiltration. **p < 0.05 compared to the Sham group.

**FIGURE 10:**
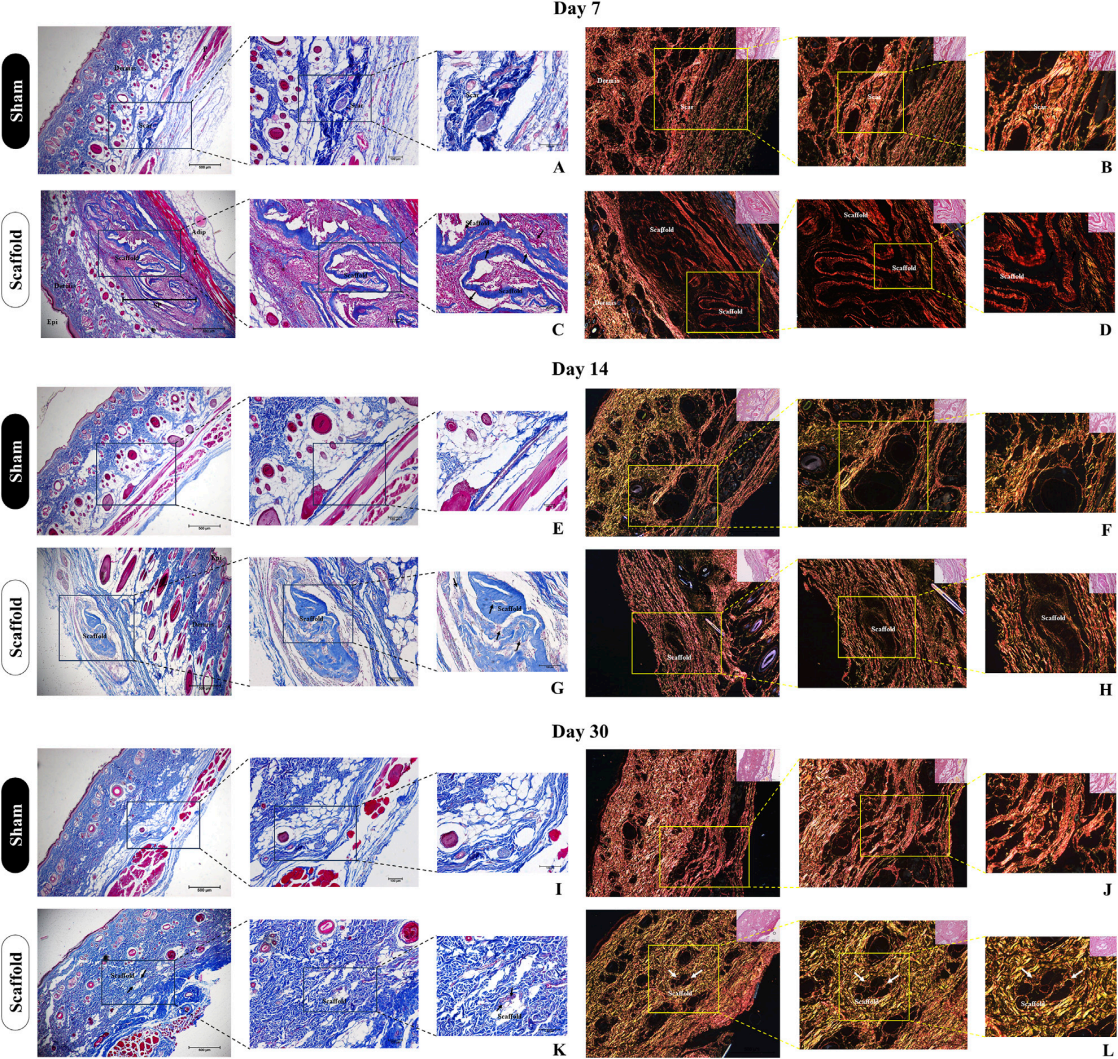
Histological evaluation of collagen deposition and biomaterial integrity after subcutaneous implantation of decellularized placental membranes in immunocompetent rats (n = 5 per group). Masson’s trichrome staining of the implanted region on day 7 **(C)**, 14 **(G)** and 30 **(K)** and their respective Sham control groups **(A,E,I)**. Black arrows indicate the scaffolds position in the tissue. Picrosirius Red staining of the implanted region on day 7 **(D)**, 14 **(H)** and 30 **(L)** and their respective Sham control groups **(B,F,J)**. White arrows indicate the scaffolds position in the tissue.

The biomaterials’ immunogenicity is a crucial factor for their use in preclinical models, as the tissue response to these materials should not incite an exaggerated inflammatory reaction or cause damage to the surrounding tissue (Almeida et al., 2024). To evaluate the biocompatibility of placental matrices, we used a subcutaneous scaffold implant model in immunocompetent Wistar rats (Figures 9, 10). A clinical evaluation was performed throughout the experimental period, revealing no signs of edema, redness, or infection, suggesting that the scaffold did not induce any excessive inflammatory response. Histopathological analysis using H&E staining at 7 days post-implantation revealed that the scaffold remained intact, surrounded by a local inflammatory response with a substantial recruitment of inflammatory cells (Figure 9G). This was expected due to the presence of a foreign body and the trauma caused by the surgical procedure to implant the scaffold (Figure 9B). However, by day 14 post-implantation, the inflammatory response had diminished, with no further leukocytic infiltration observed, as compared to day 7 (Figure 9D). No signs of vacuolization, edema, or tissue degradation were observed around the implant, and the scaffold showed signs of color loss, indicating biomaterial degradation and integration with the host tissue. This observation was further supported by the analysis at 30 days post-implantation, where only degraded remnants of the scaffold were left in the subcutaneous tissue, demonstrating that the biomembrane had been degraded and absorbed by the surrounding tissue without signs of immune rejection or tissue necrosis (Figure 9F).

These findings were further complemented by histological analysis using Masson’s trichrome staining, highlighting the total collagen content in the sample and the surrounding tissue (Figure 10). It is evident that as the subcutaneous implantation period progresses, the scaffold gradually adopts a lighter blue hue, indicating its degradation by the host tissue cells (Figures 10C,G,K). This observation is reinforced by the analysis of the predominant collagen fiber types in the scaffold through polarized light microscopy of Picrosirius Red staining (Figure 10). At 7 days post-implantation, the placental scaffolds display a reddish coloration, which indicates the presence of dense, mature, and still-structured collagen (Figure 10D). By day 14, a significant reduction in birefringence is observed, with the red coloration of the fibers shifting to yellowish and greenish types (Figure 10H), indicating continued degradation of the scaffold. At 30 days, the collagen fibers remaining from the scaffold are almost undetectable, deeply embedded within the adjacent tissue, which further corroborates the previous histopathological findings (Figure 10L). These results suggest that acellular porcine placental biomembranes are both biocompatible and biodegradable, as there is no evidence of an exaggerated immune response, and the near-complete disappearance of the scaffold indicates its absorption by the host’s adjacent tissue.

## 4 Discussion

The bioactive properties of placental tissues for regenerative medicine applications directly reflect their multiple roles in fetal survival and pregnancy maintenance. From modulating the endometrial microenvironment to the selective transfer of metabolites and biomolecules to the fetus, the placenta employs mechanisms that make it a unique and multifunctional organ (Burton and Fowden, 2015; Burton and Jauniaux, 2015; Khorami-Sarvestani et al., 2024). The placental ECM plays a key role in these properties, as it not only preserves tissue memory, but also contributes to the unique biomechanical properties of the tissue, serving as a reservoir of cytokines, growth factors, and other biomolecules with immunomodulatory and antimicrobial activities. These biological scaffolds provide critical molecular cues for cell adhesion, proliferation, and differentiation (Choi et al., 2013). Studies have demonstrated that human placental matrices are capable of supporting the culture of various cell types, as well as inducing and enhancing the differentiation of mesenchymal stem cells (MSCs) into osteogenic and chondrogenic lineages (Khosrowpour et al., 2023; 2024). In terms of *in vivo* applications as biological dressings, several studies have proven the effectiveness of scaffolds, hydrogels, and hybrid biocomposites derived from decellularized human placental tissues as biocuratives for complex wounds, including infected wounds, diabetic ulcers, burn-induced injuries, and chronic ulcers (Gholipourmalekabadi et al., 2018; Mirzadegan et al., 2022; Wali et al., 2022).

In light of this, additional studies involving placental scaffolds from bovine, murine, and canine sources have also yielded significant results both *in vitro* and *in vivo*. These studies have demonstrated that such matrices are capable of supporting cells from several tissue origins and species, highlighting their translational potential for a wide range of applications (Bektas et al., 2024; Fratini et al., 2018; Matias et al., 2018). A preliminary study using murine placental matrices cultured with murine embryonic stem cells (mESCs) showed that the scaffold not only supported the differentiation of the cells into hepatic-like cells, but also that the biomimetic construct exhibited structures similar to hepatic parenchyma (Da Silva Nunes Barreto et al., 2023). In our study, aiming to develop and validate a placental scaffold for multispecies applications, we produced acellular biomembranes derived from porcine allantochorion for potential use as biocuratives in tissue repair. Decellularized biomaterials derived from porcine placentas offer several advantages over the use of placentas from other animal species for the production of bioproducts for regenerative medicine. Regarding the amount of material available, rodent and lagomorph placentas are small, and the quantity of decellularized material is minimal, which hinders large-scale production (Favaron et al., 2019). In contrast, placentas from large animals such as cattle present other limitations. The gestation period for a calf is approximately 283 days, resulting in only one placenta per birth (Ballesteros et al., 2020). Another drawback of bovines is their natural behavior, cows often give birth in open pastures and tend to consume the placenta post-delivery, which compromises the quality of the harvested placental material (Mota-Rojas et al., 2020). Moreover, the decellularization process for bovine placentas is significantly more complex and demands more sophisticated equipment. Previous studies have employed perfusion-based methods to decellularize bovine placentomes, which, although preserving structural integrity, led to degradation of key extracellular matrix (ECM) proteins, reducing the overall quality of the resulting scaffold (Barreto et al., 2018). When considering placentas from domestic carnivores such as dogs, the lack of availability and standardization of samples alone makes their widespread use unfeasible (Matias et al., 2018) As for human placentas, although there are already commercial products derived from human placental matrices, this raw material also presents limitations, including the requirement for informed consent, meticulous biosafety testing, religious drawbacks and the inherent restriction of obtaining only one placenta per pregnancy, especially relevant in the context of declining birth rates, particularly in developed countries where most major bioproduct companies are based (Protzman et al., 2023). Based on this, the choice of porcine tissue was motivated by several favorable factors. Pigs are livestock animals with a rapid reproductive cycle and produce a large number of placentas per delivery, typically between 8 and 12. The biosafety of the material is also highly reliable, as swine meat production is regulated by stringent standards for animal feed, welfare, housing, and management (Almeida et al., 2023). In the case of Brazil, the country is one of the largest global producers and exporters of pork, ensuring a vast and steady supply of material for large-scale production of porcine placental bioscaffolds (Almeida et al., 2023). Additionally, the decellularization process itself is relatively quick, reproducible, requires few reagents and commonly available laboratory equipment, and yields a high volume of decellularized material. Unlike most studies that utilize the amniotic membrane for scaffold production, we chose the allantochorion for its distinctive properties. It is composed of thicker tissue, enriched with a higher concentration of extracellular matrix, is vascularized, and lies in close proximity to the maternal endometrium. This membrane is actively involved in maternal-fetal communication, immune modulation, and serves as a reservoir of cytokines and growth factors, making it a highly angiogenic, proliferative, and immunologically privileged site (Johnson et al., 2021).

Our data showed that the produced biomembranes maintained their structural and compositional integrity, directly reflecting their bioactivity. Both fibrous components, such as collagen I, III, and elastin, as well as non-collagenous elements like glycosaminoglycans (GAGs) and adhesive glycoproteins such as fibronectin and laminin, are crucial for cell adhesion and migration (Doryab and Schmid, 2022; Guo et al., 2012; Khalili and Ahmad, 2015). This integrity was also reflected in the biomechanical properties of the membranes, which maintained the stiffness and mechanical strength typical of native tissues. In this regard, the ECM provides mechanical signals through mechanotransduction, promoting cell adhesion and migration, modulating cytoskeletal dynamics, and influencing cellular processes such as proliferation and differentiation (Martino et al., 2018; Saraswathibhatla et al., 2023). This resistance and plasticity are crucial for biocuratives and biomembranes, enabling the repopulation of the damaged area by surrounding tissue without triggering fibrotic foci (Chen et al., 2021; Moore et al., 2015). Moreover, placental ECM components have demonstrated antifibrotic properties, particularly those associated with hyaluronic acid and reticulin (type III collagen), which are closely linked to the downregulation of the TGF-β1 pathway (Berdiaki et al., 2024; Zhu et al., 2020). Raman spectroscopic and histological analyses revealed that hyaluronic acid (HA), typically detected around the 1,067 cm^−1^ band (Essendoubi et al., 2016), were successfully preserved in the decellularized placental scaffolds. Glycosaminoglycan molecules, which are particularly vulnerable to degradation during the decellularization process in numerous studies, were also preserved, highlighting the effectiveness of our methodology in safeguarding even the most sensitive components of placental extracellular matrix (Uhl et al., 2019).

The preservation quality of the placental ECM components directly influenced its capacity to support cellular adhesion and viability, as both cell types employed in this study exhibited robust cell–ECM interactions and high survival rates throughout a 10-day culture period on the scaffold. Given that the scaffold is of porcine origin and the cells were derived from murine and canine sources, these results suggest that the biomembrane possesses the versatility to support cells from different species. This behavior is consistent with that reported for human placental-derived scaffolds, which have demonstrated broad applicability across diverse tissue environments and preclinical models, including rodents and pigs, thereby reinforcing the translational relevance and adaptability of the material (Jiang et al., 2021; Mao et al., 2022; Wetzell et al., 2024). Although the results are promising, the absence of cytocompatibility testing with human cells represents a limitation that could be addressed in future studies to strengthen the applicability of the findings, as demonstrated in previous works where human cell lines were employed to validate placental ECM-derived biomaterials performance (Somuncu et al., 2019).

Regarding the biomembranes biocompatibility, the results showed that the scaffolds did not display long-term immunogenicity and were biodegraded by the end of the analyzed period. This outcome is likely attributed to two main factors: the placental tissues intrinsic immunocompatibility and the scaffolds structural integrity. Placental tissues inherently possess mechanisms for immune evasion and modulation, particularly in hemochorial placentas, which erode the endometrium for conceptus implantation (Genest et al., 2023). The placentas used in our study are epitheliochorial, meaning there is no degradation of the endometrium or decidualization. Instead, the allantochorion is closely positioned with the maternal endometrial epithelium (Johnson et al., 2021; Seo et al., 2020). However, intense immune modulation occurs, varying with the stage of pregnancy, ensuring the viability of both the placental tissue and the conceptus throughout gestation (Velez et al., 2024). This cascade of biomolecules throughout pregnancy enhances the placental microenvironment and, consequently, the extracellular matrix (ECM), contributing to its bioactivity and non-immunogenicity (Mathew et al., 2016; McEwan et al., 2009). Uterine NK cells, in particular, play a key role in ensuring immunoprotection for the conceptus, while the synthesis of pro-angiogenic molecules promotes intense vascularization, facilitating the exchange of molecules between the mother and fetus (Sojka et al., 2019; Velez et al., 2024; Yu et al., 1993). Another key characteristic of placental tissue that may contribute to the biocompatibility of porcine placental scaffolds is its low expression of MHC class I and II molecules. This reduced presence of these molecules makes the tissue less recognizable by the immune system, potentially enhancing its immunological tolerance (Tersigni et al., 2020). Another relevant mechanism is the presence of HLA-G in placental tissue, which is associated with immune tolerance (Zhuang et al., 2021). This is important because, even after decellularization, traces of residual MHC molecules remain embedded in the ECM, which could trigger inflammatory rejection mechanisms (Kasravi et al., 2023). Therefore, porcine placental scaffolds, which have low levels of these molecules and predominantly contain HLA-G, are non-immunogenic (Ramsoondar et al., 1999). Regarding the scaffold’s integrity, studies have shown that efficiently decellularized but degraded scaffolds can trigger exacerbated immune reactions and rejection mechanisms similar to xenografts, as degraded ECM fragments act as DAMPs, which can activate dendritic cells, T lymphocytes, and the release of inflammatory cytokines (Kasravi et al., 2023; Wu et al., 2022). Cytokines such as TNF-α, IL-1β, IL-6, and IFN-γ recruit M1 macrophages, which are involved in graft rejection (Kasravi et al., 2023). While some studies have reported immunogenic responses associated with non-collagenous ECM proteins (Li et al., 2019), particularly in xenogeneic or improperly processed materials, our findings did not indicate any such response. This may be attributed to the preservation of native protein conformation and the efficiency of our decellularization method in removing immunogenic cellular residues without compromising ECM integrity. These results highlight the potential of our biomaterial to retain critical ECM components without eliciting adverse immune reactions. Based on the evidence presented, it can be inferred that the acellular biomembranes derived from decellularized porcine allantochorion preserved their structural and compositional integrity, exhibiting excellent cytocompatibility. They facilitated cell adhesion and supported high cell viability rates. Furthermore, the scaffolds demonstrated biocompatibility in a subcutaneous implantation study, not inducing any adverse immune response. These findings provide compelling evidence that this biomaterial has substantial potential for future biomedical applications.

## 5 Conclusion

This study developed extracellular matrix-based biomembranes derived from decellularized porcine allantochorion, showing a satisfactory accelularity profile and a well-organized ECM with preserved composition and mechanical properties, similar to those found in native allantochorion. The membranes also demonstrated strong biocompatibility, supporting the growth of different cell types, underscoring their potential for applications involving cells from diverse origins and species. The biomembranes were biocompatible *in vivo*, not inducing undesirable tissue responses following subcutaneous implantation. These findings suggest that porcine placental biomembranes possess the necessary characteristics to serve as biodressings for tissue repair and other applications in tissue engineering.

## Data Availability

The original contributions presented in the study are included in the article/supplementary material, further inquiries can be directed to the corresponding author.
